# CAR-T cell-derived exosomes: a new perspective for cancer therapy

**DOI:** 10.1186/s13287-024-03783-4

**Published:** 2024-06-18

**Authors:** Farnaz Sani, Shabnam Shojaei, Seyed Amirhossein Tabatabaei, Mohammadhossein Khorraminejad-Shirazi, Mona Latifi, Mahsa Sani, Negar Azarpira

**Affiliations:** 1 Shiraz Institute for Stem Cell & Regenerative Medicine, Shiraz University of Medical Sciences, Shiraz, Iran; 2 School of Medicine, Shiraz Shiraz University of Medical Sciences, Shiraz, Iran; 3 Department of Pathology, School of Medicine, Shiraz University of Medical Sciences, Shiraz, Iran; 4 Student research committee, Shiraz University of Medical Sciences, Shiraz, Iran; 5 Department of Pathology, School of Medicine, Jahrom University of Medical Sciences, Jahrom, Iran; 6 Department of Physiological Science, College of Veterinary Medicine, Oklahoma State University, Stillwater, OK USA; 7 Department of Tissue Engineering and Applied Cell Sciences, School of Advanced Medical Sciences and Technologies, Shiraz University of Medical Sciences, Shiraz, Iran; 8 Transplant Research Center, Shiraz University of Medical Sciences, Khalili Street, P.O. Box: 7193711351, Shiraz, Iran

**Keywords:** CAR-T cell, Exosome, Cancer, Cell therapy, Solid tumor

## Abstract

Chimeric antigen receptor (CAR)-T cell adoptive immunotherapy is a promising cancer treatment that uses genetically engineered T cells to attack tumors. However, this therapy can have some adverse effects. CAR-T cell-derived exosomes are a potential alternative to CAR-T cells that may overcome some limitations. Exosomes are small vesicles released by cells and can carry a variety of molecules, including proteins, RNA, and DNA. They play an important role in intercellular communication and can be used to deliver therapeutic agents to cancer cells. The application of CAR-T cell-derived exosomes could make CAR-T cell therapy more clinically controllable and effective. Exosomes are cell-free, which means that they are less likely to cause adverse reactions than CAR-T cells. The combination of CAR-T cells and exosomes may be a more effective way to treat cancer than either therapy alone. Exosomes can deliver therapeutic agents to cancer cells where CAR-T cells cannot reach. The appropriate application of both cellular and exosomal platforms could make CAR-T cell therapy a more practicable treatment for cancer. This combination therapy could offer a safe and effective way to treat a variety of cancers.

## Introduction

Cancer immunotherapy has become a major treatment option in recent years. One of the most promising approaches is Chimeric antigen receptor (CAR)-T cell therapy, which involves engineering T cells to target specific cancer cells. However, CAR-T cell therapy can also have some adverse side effects, such as cytokine release syndrome (CRS) [1]. CRS is characterized by a rapid and excessive cytokine release, leading to a cytokine storm. The cytokine storm occurs when an overwhelming amount of pro-inflammatory and inflammatory cytokines, such as Interleukin-2 (IL-2), soluble IL-2 receptor α (soluble IL-2Rα), Interferon-gamma (IFN-γ), tumor necrosis factor-alpha (TNF-α), Interleukin-6 (IL-6), soluble IL-6R, and Granulocyte-macrophage colony-stimulating factor (GM-CSF) are released into the bloodstream [2, 3]. The cytokine storm triggers immune responses, causing widespread inflammation and activation of immune cells. This immune hyperactivation can attack vital organs, including the lungs, liver, kidney, heart, brain, and central nervous system [4].

Exosomes are a type of extracellular vesicle that is secreted by many cells in the body. They have been shown to play a role in intercellular communication and may also be able to deliver therapeutic cargo to cancer cells [5]. Using CAR-T cell-derived exosomes as a therapeutic approach may provide more control over the treatment process and help reduce the risk of adverse side effects. Exosomes are a promising new point of research in cancer immunotherapy, and they can potentially revolutionize how cancer is treated. This review focuses on the therapeutic potential of CAR-T cells and CAR-T cell-derived exosomes in cancer models.

## Overview of exosomes (contents, biogenesis and uptake)

The classification of EVs falls into two major categories: ectosomes and exosomes. Ectosomes form by outward budding of the plasma membrane and forming microvesicles with a size range of ~ 50 nm to 1 μm, and as for exosomes with the endosomal origin, they have a size range of 40 nm to ~ 160 nm (~ 100 nm in average) [6]. Over the years, it has been determined that these two exosome classifications have physiological and pathological roles. But in the last decade, mounting evidence has shown that membrane vesicles are important messengers in intercellular communication. Exosomes have been the most studied of these vesicles and are well-characterized [7–9].

**Fig. 1:**
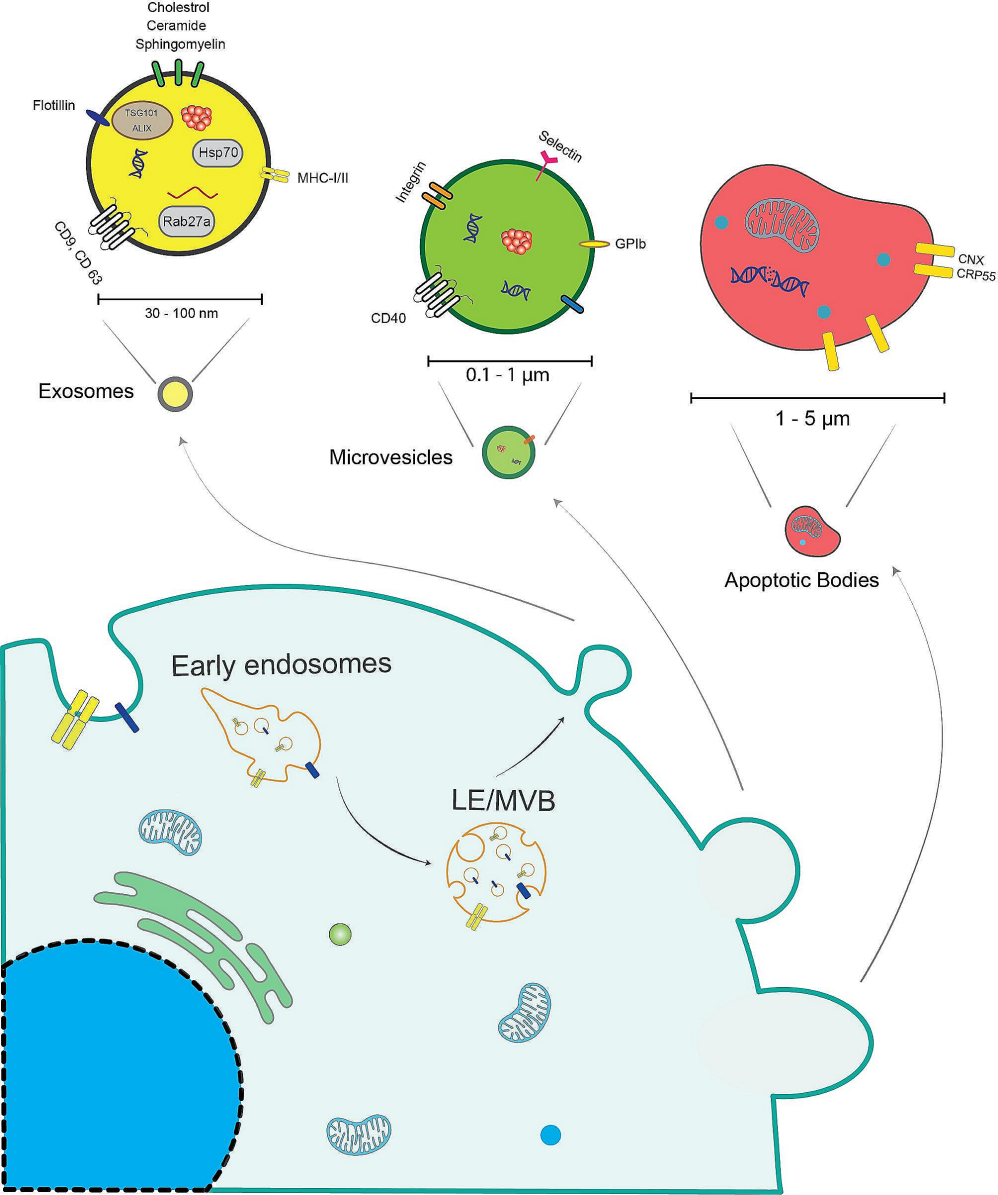
Exosomes, microvesicles, and apoptotic bodies are different types of extracellular vesicles (EVs) that play important roles in intercellular communication. Exosomes are small vesicles formed through inward budding of the endosomal membrane, while microvesicles are larger vesicles formed through outward budding of the plasma membrane. Apoptotic bodies are vesicles released during programmed cell death. These EVs carry various molecules and can be taken up by neighboring cells, facilitating communication and transfer of biological material

The exosomes form by double invagination of the plasma membrane. First, invagination makes a cup-shaped structure that starts with early sorting endosomes (ESEs), which mature into late sorting endosomes (LSEs) by the contribution of the trans-Golgi network and endoplasmic reticulum, and later, inward invagination of the endosomal membrane forms multivesicular bodies (MVBs) containing intraluminal vesicles (ILVs, future exosomes). The exosomes will finally be released by the fusion of MVB with the plasma membrane (Fig. 1) [10–12].

Exosomes are heterogeneous due to their size differences, content, cellular origin, and function in the recipient cell. Their size heterogeneity comes from the uneven invagination of exosomal content in MVBs [12, 13]. The exosomal contents can vary due to their function; usually, exosomes have the following cargo: membrane proteins, cytosolic and nuclear proteins, as well as extracellular matrix and nucleic acids, such as mRNA, noncoding RNA species, and DNA [14, 15]. Their role and effect on recipient cells can also be different due to cell surface receptors and can induce cell survival, apoptosis, and immunomodulation. Regarding exosomes, heterogeneity can also result from their cell origin [12].

Exosome uptake is connected to multiple mechanisms, such as clathrin-dependent endocytosis, phagocytosis, and micropinocytosis [16, 17]. Exosomes possess multiple surface molecules that enable them to interact with numerous recipient cells. This means that exosomes can either transmit multiple signals with variable intensity to different cells or have no physiological function on cells and only act as multipliers of nutrient levels [7].

## Biological functions of exosomes

As discussed before, exosomes have both physiological and pathological properties. They can carry a variety of molecules, including microRNAs, mRNAs, and proteins. Exosomes can transfer these molecules to other cells, which can then use them to change their behavior. This process is called exosome-mediated intercellular communication [18]. Exosomes can communicate with cells in the immediate vicinity or travel through the bloodstream to communicate with cells in other parts of the body. Exosomes can even pass through the blood-brain barrier, which separates the blood from the brain [19]. It was reported that exosomes are a versatile way for cells to communicate with each other. They can deliver multiple signals simultaneously, and they can target multiple cells and multiple locations. This makes exosomes a powerful tool for intercellular communication. Exosomes are a rapidly emerging field of research. Scientists are still learning about their many functions. However, it is clear that exosomes play an important role in intercellular communication and many other biological processes [20].

## Overview of using CAR-T cells

The U.S. Food and Drug Administration approved CAR-T cell therapy as the first gene therapy [21]. Genetically engineered T cells expressing a chimeric antigen receptor (CAR) are rapidly emerging as a promising new treatment for hematological and non-hematological malignancies. In solid tumors, CAR-T therapy has not achieved the clinical success that has been observed in hematological malignancies. CAR-based adoptive immunotherapies, which use genetically modified T lymphocytes to provide both tumor targeting and immune responses, can act as living drugs that exert constant cytotoxic attacks on targeted cells. CAR-T cells and the replication of these cells in vivo can boost cytokine release, which is not suitably controllable. This effect is a potential source of adverse effects, such as CRS, the cytokine storm, and on-target, off-tumor responses [5, 22].

### Solid tumor cancer

CAR-T cell therapy has revolutionized the field of cancer treatment, particularly in the management of hematological malignancies. However, its application in solid tumors has presented unique challenges due to the complexities of the tumor microenvironment, such as heterogeneity and the hostile tumor microenvironment. Advancements in target identification, enhancing tumor infiltration, managing toxicities, and exploring combination therapies offer hope for the effective use of CAR-T cell therapy in combating solid tumors and bringing us closer to personalized and precise cancer treatments [23–26].

Target selection is indeed a significant obstacle to CAR-T cell therapy for solid tumors. The complex nature of solid tumor tissues and the variability in protein expression profiles among different tumor cells make it challenging to identify targets that can effectively cover all tumor cells. Even when antigen-positive tumor cells are eliminated, a significant number of antigen-negative cells may persist, leading to a high rate of tumor recurrence [23, 27].

One of the difficulties in target selection for solid tumors is the fact that solid tumor cells originate from healthy tissues. Many antigens highly expressed in tumor cells are also expressed in small amounts in normal cells. This poses a challenge in avoiding the nonspecific killing of healthy cells, resulting in on-target, off-tumor side effects. Tumor heterogeneity is another significant challenge in CAR-T cell therapy for solid tumors. Solid tumors often exhibit high levels of heterogeneity, meaning that different tumor cells within the same patient may not express the same tumor-associated antigen. Therefore, if a single target is recognized by CAR-T cells for treating a solid tumor, the success of the therapy may depend on the heterogeneity of the tumor [28, 29].

CD133, a novel tumor stem cell marker, has been identified as an overexpressed target in many solid tumors and is being explored as a target for CAR-T cell therapy. However, the high heterogeneity of solid tumors makes their treatment much more challenging compared to hematological malignancies. The critical challenge lies in the selection of appropriate targets that can effectively address the heterogeneity and complexities of solid tumors [24].

CAR-T cells have been observed to return to the lymphatic and blood systems, increasing the likelihood of encountering blood tumor cells. The success of CAR-T cell therapy in solid tumors depends on their ability to penetrate tumor tissue through vascular endothelial cells [29].

The limited migration and invasion of CAR-T cells into solid tumors can be attributed to the dense fibrotic matrix present in the tumor microenvironment and the downregulation of chemokines that facilitate T-cell infiltration into tumor tissues [23].

The hostile immunosuppressive tumor microenvironment in solid tumors further complicates CAR-T cell therapy. The tumor microenvironment (TME) is a complex environment consisting of various extracellular matrices, stromal cells, inflammatory cells, and vasculature. In solid tumors, the TME is characterized by low vascularization, hypoxia, and a high concentration of extracellular matrix [29]. Several factors contribute to the immunosuppressive TME and pose significant challenges to CAR-T cell therapy. Solid tumors often exhibit an acidic TME due to increased glycolysis and lactate production. The low pH inhibits T cell function and promotes immune evasion by impairing CAR-T cell activity and cytokine production [30].

In solid tumors, the TME is marked by limited access to tryptophan, an essential amino acid vital for the optimal functioning of T cells. Tumor cells and immune suppressive cells, such as myeloid-derived suppressor cells (MDSCs), can consume tryptophan through the enzyme indoleamine 2,3-dioxygenase (IDO), leading to T cell suppression. Solid tumors often have regions of low oxygen levels (hypoxia) within the TME. Hypoxia can impair CAR-T cell function and survival, reducing their effectiveness in targeting tumor cells. Immune suppressive cells within the TME, such as regulatory T cells (Tregs), MDSCs, and tumor-associated macrophages (TAMs), secrete immunosuppressive molecules like transforming growth factor-beta (TGF-β), interleukin-10 (IL-10), and programmed cell death ligand 1 (PD-L1). These molecules inhibit CAR-T cell activity, suppress immune responses, and promote tumor immune escape [31–33].

These factors collectively create an immunosuppressive and hostile environment within solid tumors, hampering the efficacy of CAR-T cell therapy. Overcoming these challenges is a key focus of research and involves strategies to enhance CAR-T cell persistence, modulate the TME, and combine CAR-T therapy with other immunomodulatory approaches to improve treatment outcomes in solid tumors.

### Lung cancer therapy

Lung cancer is indeed one of the most prevalent and deadliest cancers worldwide [34–36]. Unfortunately, a large percentage of lung cancer patients are diagnosed at advanced stages when treatment options are limited, leading to low 5-year survival rates of approximately 10–20% [37]. While surgical resection combined with adjuvant therapy remains the main treatment strategy, only a fraction of patients are eligible for surgery, and recurrence after surgery remains a significant concern [38]. Therefore, there is an urgent need to explore new treatment strategies that can impede tumor progression and improve the survival outcomes for lung cancer patients [34].

CAR-T cell therapy, with its proven success in hematological malignancies, offers a new ray of hope for treating solid tumors, including lung cancer. However, it faces similar challenges as other solid tumors, as mentioned earlier. Additionally, the clinical application of CAR-T cells is hindered by challenges such as tumor lysis syndrome, neurotoxicity syndrome, and cytokine release syndrome [34, 39, 40].

Based on the information provided, there is a list of potential tumor-associated antigens (TAAs) for CAR-T cell therapy in patients with lung cancer. Notably, the selection of the optimal TAA for CAR-T cell therapy depends on various factors, including the specific characteristics of the tumor and its microenvironment. Further research and clinical trials are necessary to determine the efficacy and safety of targeting these TAAs in CAR-T cell therapy for lung cancer [41, 42]:

- **EGFR** (Epidermal Growth Factor Receptor) is a protein that is often overexpressed in solid tumors, including lung cancer and brain metastasis. EGFRvIII is a variant of EGFR that is particularly common in lung cancer. CAR-T cells that are engineered to target EGFRvIII have been shown to be effective in killing EGFRvIII-positive lung cancer cells. This could be a potential therapeutic strategy to prevent the recurrence and metastasis of lung cancer after surgery [43, 44].
- **Mesothelin** (MSLN) is a cell adhesion glycoprotein that is associated with high tumor aggressiveness and a poor prognosis in lung cancer patients. It is considered to be a desirable target for CAR-T therapy in solid tumors, including lung cancer [45, 46].
- **Mucin 1** (MUC1) is a transmembrane protein that is involved in cancer cell adhesion and metastasis. Expression of MUC1 is significantly higher in lung cancer tissues compared to normal lung tissues. It is a potential target for CAR-T cell therapy in lung cancer [37].
- **HER2** (Human Epidermal Growth Factor Receptor 2) is highly expressed in lung cancer. It facilitates the proliferation, invasion, and angiogenesis of cancer cells. HER2 can serve as a promising biomarker for the diagnosis and treatment of lung cancer [34].

### Breast cancer

Breast cancer currently holds the highest global incidence and mortality rates among cancers affecting women [47]. The emergence of resistance to existing treatments underscores the need for the development of novel therapeutics. CAR-T cell therapy, an immunotherapy approach that utilizes a patient’s immune cells to combat cancer, has shown promise and has been widely applied in hematologic malignancies. Its application in treating solid tumors, including breast cancer, has gained momentum. Within breast cancer cells, specific molecules with altered expressions have been identified as potential targets for CAR-T cell therapy. Nineteen antigens, namely HER2, EGFR, hepatocyte growth factor receptor/c- mesenchymal-epithelial transition factor (HGFR/cMET), tyrosine-protein kinase transmembrane receptor (ROR1), AXL receptor tyrosine kinase (AXL), MUC1, MSLN, CD70, CD133, CD44v6, epithelial cellular adhesion molecule (EpCAM), chondroitin Sulfate Proteoglycan 4 (CSGP4), intercellular adhesion molecule 1 (ICAM1), tumor endothelial marker 8(TEM8), trophoblast cell-surface antigen 2 (TROP2), folate receptor alpha (FRα), natural killer group 2, member D (NKG2D), GD2, and Carcinoembryonic antigen (CEA), have been investigated as targets for CAR-T cell therapy in breast cancer. Most of these antigens belong to the Receptor tyrosine kinases family and cell surface proteins. Studies have demonstrated that all 19 antigens exhibit anti-tumor effects, inhibiting tumor growth and inducing the release of proinflammatory cytokines.

Despite the progress made in CAR-T cell therapy in recent years, several challenges persist, including inadequate trafficking and infiltration, the presence of an immunosuppressive environment, a lack of tumor-specific or tumor-associated antigens, and CAR-T cell toxicities [48].

While CAR-T cell therapy has achieved significant advancements in breast cancer, its application in clinical settings is still distant due to insufficient supporting evidence. To expedite the clinical implementation of CAR-T cell therapy for breast cancer patients, exosome therapy and clinical trials are essential to address safety concerns and overcome the existing challenges.

### Hematological and non-hodgkin lymphoma (NHL) therapy

CAR-T cell immunotherapy has achieved significant success in treating hematological malignancies, with high remission rates observed in clinical trials. CAR-T cells targeting CD19 have shown long-lasting remission effects on drug-resistant B-cell malignancies, and the cure rate for relapsed and refractory acute B-lymphocytic leukemia has reached approximately 80–90% [49].

The success of CAR-T therapy in hematological malignancies, such as acute lymphoblastic leukemia and large B-cell lymphomas, has revolutionized their treatment [50]. The approval of CD19-directed CAR-T cells by the FDA for the treatment of relapsed or refractory pediatric and young adult diffuse large B cell lymphoma (DLBCL) further validates the effectiveness of this approach [29, 51].

One of the key factors contributing to the success of CAR-T cell therapy in hematological malignancies is the presence of a monoclonal disease, where a single target antigen, such as CD19, is expressed on the surface of all neoplastic cells. This enables CAR-T cells to effectively target and eliminate the cancer cells. To overcome these challenges, future treatment approaches for tumors will likely involve combining immunotherapy-based treatments, such as CAR-T cell therapy, with other treatment modalities [51].

#### NHL

The recent approval of CD19-specific CAR-T cell therapy by the FDA has provided a promising treatment for chemotherapy-resistant B-cell non-Hodgkin lymphoma (NHL) and relapsed/refractory pediatric and young-adult diffuse large B-cell lymphoma (DLBCL). CD19, a pan-B-cell marker, is highly expressed in most B-cell NHLs, making it a suitable target for CAR-T cell therapy in these malignancies. In the case of acute lymphoblastic leukemia (ALL), the clinical effect of anti-CD19 CAR-T cells appears to be more significant compared to lymphoma. This may be due to differences in disease biology and response to treatment. Patients with peripheral T-cell lymphomas, on the other hand, present a heterogeneous set of diseases with a generally poor prognosis, posing challenges for CAR-T cell therapy [52].

CAR-T cell therapy has evolved from first-generation to second-generation CAR-T cells. The first-generation CAR-T cells utilized a CD3z chain as a signaling domain, while the second-generation CAR-T cells incorporated additional co-stimulatory domains to enhance their antitumor activity. Second-generation CAR-T cell therapy has shown more significant antileukemic responses, with high recovery rates reaching up to 90% in patients with recurrent B-cell ALL. However, treatment outcomes can still be poor in individuals with high-risk characteristics, such as early relapse, refractory disease, and certain types of lymphoma [52, 53].

Currently, anti-CD19 CAR-T cells have demonstrated sustained remission rates in about 40% of chemotherapy-resistant DLBCL, high-grade B-cell lymphoma (HGBCL), and primary mediastinal B-cell lymphoma (PMBCL) patients who have not received previous treatment options. These products are also being used in patients with aggressive lymphoma who have relapsed after at least two prior lines of treatment. The advancements in CAR-T cell therapy have provided highly effective solutions for treating patients with NHL.

### Prostate cancer

While limited studies have been conducted using CAR-T cell therapy in metastatic prostate cancer (mPCa), there have been investigations into potential targets for CAR-T cell therapy in this context. Two important candidates for CAR-T cell-targeted antigens in mPCa are prostate-specific membrane antigen (PSMA) and prostate stem cell antigen (PSCA). In vitro and in vivo models have demonstrated that PSMA-CAR-T cells can proliferate and recognize PSMA-positive cells. Second-generation CAR-T cells have shown improved killing effects compared to previous generations, representing a novel immune-targeted approach for mPCa [54–56].

In prostate cancer (PCa), several proteins have been identified as preferentially expressed by malignant cells, including prostate-specific antigen (PSA), prostatic acid phosphatase (PAP), prostate stem cell antigen (PSCA), T-cell receptor gamma alternate reading frame protein (TARP), transient receptor potential (trp)-p8, and prostate-specific membrane antigen (PSMA). Recently, various studies have explored these prostate tumor-associated antigens (TAAs) as targets to induce an immunological response in PCa patients [51].

Overall, the use of CAR-T cell therapy in metastatic prostate cancer is an area of active research, and while promising preclinical results have been obtained, further investigation is required to assess the safety and effectiveness of this approach in clinical settings.

### Glioblastoma

The use of exosomes holds promise for the application of CAR-T cell technology in solid tumor therapy, including targeting tumors in specific regions such as glioblastoma (GBM). While the exploration of CAR-T cell therapy in GBM is in its early stages, initial results have shown feasibility, safety, and even signs of efficacy using this approach [57].

There are several challenges that need to be addressed in the further development of CAR-T cell therapy for GBM. These challenges include enhancing CAR T-cell infiltration into tumors, optimizing infusion dosing and frequency, modulating the immunosuppressive tumor microenvironment, and addressing the molecular heterogeneity that is inherent to GBM. Additionally, there are general challenges associated with immunotherapy in GBM, such as managing concomitant steroid use, distinguishing true tumor progression from radiographic pseudo-progression, and identifying potential biomarkers of response. These challenges need to be considered and addressed in any CAR-T cell study for GBM. The initial CAR targets for GBM, such as IL-13 Rα2, EGFRvIII, and HER2, are just a few examples of the potential antigens being explored for CAR targeting in this disease. Clinical investigations are already underway for CAR-T cells targeting ephrin-A2 and EGFR, and novel antigens like CD70 have recently been discovered. Results from these studies and others are eagerly anticipated to further advance our understanding of CAR-T cell therapy for GBM [57, 58].

In summary, while there are challenges to overcome, early results have shown promise for CAR-T cell therapy in GBM. Ongoing clinical investigations and the exploration of novel antigens as CAR targets provide hope for improved therapies for this challenging disease.

### CAR T-cell and oncolytic virotherapy

CAR-T cells and oncolytic viruses (OVs) have shown potential for targeting both hematologic and solid tumors. However, as monotherapies, their efficacy has been limited, particularly in the case of solid tumors. To address this challenge, the field of cancer gene therapy has been focusing on developing combination treatment strategies that involve chemotherapy, radiotherapy, and other immunotherapies like immune checkpoint inhibitors (ICIs) [59].

The combination of CAR-T cells and OVs may improve outcomes for patients with various types of cancer. By integrating the strengths of both approaches, there is potential for enhanced tumor targeting, immune activation, and therapeutic efficacy [60–62].

However, further research and clinical trials are needed to assess the safety, feasibility, and effectiveness of these combination strategies in a wider range of cancer types and patient populations.

### Limitations of CAR-T cell therapy

Indeed, there are several challenges that need to be addressed to fully realize the potential of CAR-T cell therapy. Some of these challenges include understanding the tumor microenvironment. The host tumor microenvironment plays a critical role in modulating immune responses and can impact the effectiveness of CAR-T cell therapy [63]. The other challenge is expanding the applicability of CAR-T therapy because CAR-T cell therapy needs to be made available for the treatment of a wider range of human cancers. This involves identifying and testing novel antigens and optimizing CAR designs to target specific types of cancer. Also, CAR-T therapy is currently expensive and can be cost-prohibitive for many patients. Addressing the pricing and affordability of CAR-T cell therapy is essential to ensuring broader access and availability [64]. Managing side effects is another issue; CAR-T cell therapy can be associated with side effects such as CRS, high fever, sinus tachycardia, hypotension, hypoxia, neurotoxicity, depressed cardiac function, other organ dysfunction, and graft-versus-host disease (GVHD). Strategies to manage these side effects need to be developed to improve patient safety and treatment outcomes [65, 66].

CAR-T cells can experience exhaustion and senescence, leading to diminished function and reduced effectiveness of the therapy. Understanding and addressing T cell exhaustion is crucial for maximizing the long-term efficacy of CAR-T cell therapy [67].

Enhancing response to immune checkpoint blockade is important in cancer therapies. Exhausted T cells can dampen effector immunity and reduce responsiveness to immune checkpoint blockade therapies. Checkpoint blockade therapy has emerged as a significant breakthrough in cancer treatment by targeting immune checkpoints that regulate T cell responses. One study exploring the importance of immune checkpoint blockade and the role of exhausted T cells in dampening effector T cell [68]. This study focused on investigating the relationship between exhausted T cells, immune checkpoint blockade targeting programmed cell death protein 1 (PD-1), and the generation and maintenance of aggressive cancer stem cells (CSCs). CSCs are a small population of cells within tumors that possess self-renewal and tumor-initiating capabilities, contributing to tumor growth and therapy resistance. The authors demonstrated that terminally exhausted CD8 + T cells, which are unresponsive to PD-1 blockade, play a significant role in promoting the generation and maintenance of aggressive CSCs. They found that the presence of exhausted CD8 + T cells in the tumor microenvironment led to increased expression of immune checkpoint molecules, such as PD-L1, on CSCs. This interaction between exhausted T cells and CSCs resulted in the suppression of effector T cell activity and enhanced CSC stemness features [68].

Strategies to overcome T cell exhaustion and enhance the response to immune checkpoint inhibitors need to be explored to improve the outcomes of combination therapies [69, 70].

Addressing these challenges will require ongoing research, innovation, and collaboration among scientists, clinicians, and industry partners. Despite these challenges, the increasing knowledge about CAR-T cell therapy provides valuable insights that can guide further improvements and advancements in the field.

## Advantages of CAR-T cell-derived exosomes

CAR-T-cell therapy presents limitations, including intense toxicities, antigen escape, and limitations of tumor infiltration. However, CAR-T-cell-derived EVs offer a safer and potentially efficacious immunotherapy option, garnering interest in cancer research. These exosomes can be customized to carry various cargoes, such as receptors of tumor-targeting, small interfering RNAs (siRNAs), or cytokines, that enhance the effects of anti-cancer. Notable benefits of CAR T-cell-derived extracellular vesicles (EVs) include safety (cell-free nature), modification ease, feasible detection, and specification of cancer [71, 72]. This will be further explained in the following paragraphs and depicted in Table 1.

**Table 1:** Pros and cons of CAR-T cells and CAR-T cell-derived exosomes

Phenomenon	CAR-T cell	CAR-T cell derived exosomes	References
Cytokine releasing syndrome	++	-	[5, 58, 72, 118, 119]
Direct attacker	+	++	[5, 72, 118]
“Off-the-shelf “therapy	+	++	[5]
Cross biological barriers	-	++	[5, 72, 118]
Neurotoxicity	++	-	[5, 58, 72, 118, 119]
Penetration deep tumor tissue	+	++	[5, 72]
PD-L1 Immunosuppression	+	-	[5, 71, 119]
Adapt to the complexities and changes of cancer	+	++	[90, 91]

### More controllable conditions

One of the most critical side effects related to the infusion of CAR-expressing T cells is the CAR-T cell-induced CRS [73, 74]. The symptoms of CRS include nausea, headaches, tachycardia, hypotension, rash, and shortness of breath. These symptoms are brought on by the immune cells’ secretion of cytokines. A cytokine storm is a severe type of CRS that frequently results in hypotension and high fever, which may eventually lead to organ failure or even death [73, 74]. Nearly two-thirds of CAR-T cell patients experience CRS, which usually appears 10 days following cell infusion [74, 75]. In the initial CAR-T cell clinical trials, there were two reported fatalities [76, 77]. This kind of potentially fatal consequence is mostly related to CAR-T cells’ uncontrolled secretion of cytokines. First, a systemic inflammatory response resembling sepsis is brought on by the release of cytokines from a large number of infused antitumor lymphocytes (up to 1011) [74]. The recipient’s life may be in danger if a cytokine storm causes arrhythmia, cardiac arrest, hepatic failure, or renal failure. Second, CAR-T cells can expand out of control and behave as “living drugs” that can cause CRS. In addition to eliciting a potent cytokine response, the effect of a costimulatory receptor like CD28, 4-1BB, DPA10, OX40, or ICOS on the structure of second- and third-generation CARs significantly improves absolute T-cell proliferation after repeated antigen exposure [78]. In patients with chronic lymphocytic leukemia, for instance, the 4-1BB-incorporated CD19 CAR-T cells can proliferated more than 1000-fold following delivery [79]. The in vivo expansion of CAR-T cells is thought to be essential and to be related to both toxicity and response. Steroids, vasopressors, and IL-6 blocking, as well as supportive treatment administered in the critical care unit, have all been utilized successfully to address CRS-related problems [80]. In contrast to traditional medication-induced side effects, CAR-T cell-induced toxicity cannot be controlled by just reducing the dosage of the medicine. Researchers have attempted to include suicide genes in CARs as a safety switch to eliminate CAR-T cells in the event of extreme toxicity [81]. This strategy involves engineering CAR-T cells with suicide genes that can be activated to induce cell death when adverse effects occur. Including this safety switch, allows for the timely and controlled elimination of CAR-T cells, minimizing potential harm to patients.

**Fig. 2:**
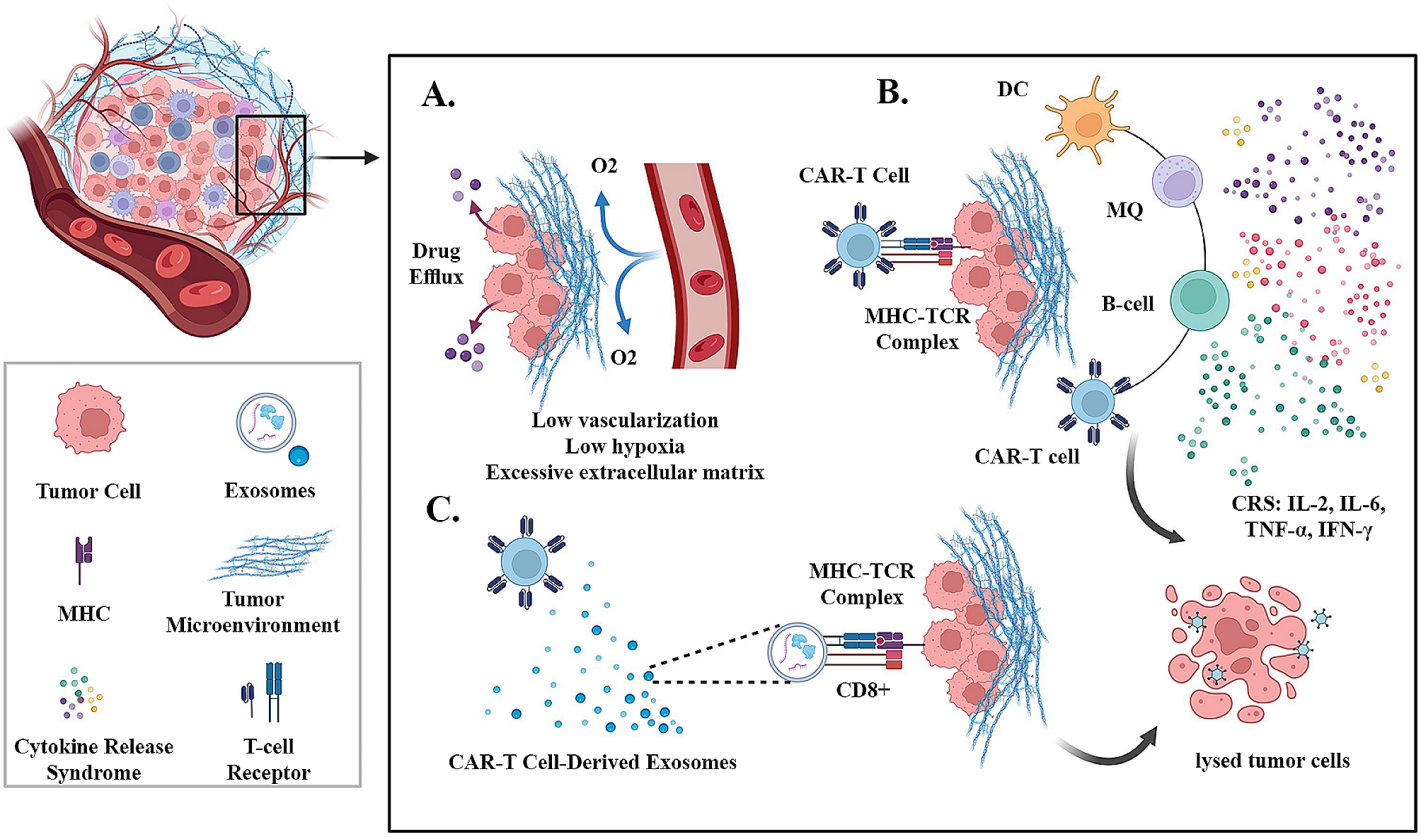
**A.** The tumor microenvironment (TME) is distinguished by limited blood vessel formation, low oxygen levels (hypoxia), and an abundance of extracellular matrix components. Hypoxia can impair CAR-T cell function and survival, reducing their effectiveness in targeting tumor cells. **B.** Upon recognition of tumor antigens, CAR T-cells initiate an immune response by releasing inflammatory cytokines. This triggers immune cells to produce a large quantity of cytokines, leading to a condition known as cytokine release syndrome (CRS). CRS is primarily a systemic inflammatory reaction caused by the release of numerous inflammatory factors from activated immune cells, including T cells, macrophages (MQ), B cells, monocytes, natural killer cells, and dendritic cells (DC). Clinical manifestations of CRS encompass fever and dysfunction of multiple organs. **C**. Exosomes share similar properties to CAR-T cells, making them a potential tool for controlling CAR-T cell-induced toxicity by directly targeting tumors and potentially replacing the need for immune cells. These exosomes contain surface membrane molecules found on T cells, such as T cell receptor (TCR) and CD8, enabling them to selectively target and eliminate tumor cells. Upon binding to a tumor cell, the TCR on the exosome interacts with the tumor cell’s antigen/MHC complex, leading to the formation of a conjugate between the exosome and the tumor cell. This conjugate delivers a lethal blow to the tumor cell, resulting in its death

CAR-T cell-derived exosomes have the potential to be used as a direct attack against cancer cells, making these “living drugs” more controllable. This is because exosomes have the same biologic properties as CAR-T cells. CAR-T cell-derived exosomes can be used to control CAR-T cell-induced toxicity by attacking tumors directly and replacing the need for immune cells (Fig. 2). The preliminary research by Zitvogel and associates provides this theory [82]. In their in vivo investigation, exosomes generated by mouse DCs pulsed with tumor peptides caused the rejection of existing tumors. In vitro, the exosomes generated by DCs were just as effective in inducing antitumor immune responses as cells with a similar amount of DCs (between 0.5 × 106 and 1 × 106 DCs). Exosomes produced by human T cells have been proven to be crucial in the interaction between cytotoxic T lymphocytes (CTL) and their target cells in early research by Peters et al. [83]. The surface membrane molecules of CTLs, such as T cell receptor (TCR), CD3, and CD8, are present in CTL-derived exosomes. These molecules allow the exosomes to specifically target and kill tumor cells. When the exosomes bind to a tumor cell, the TCR on the exosome interacts with the tumor cell’s antigen/MHC complex. This interaction triggers the formation of a conjugate between the exosome and the tumor cell. The conjugate then delivers a lethal hit to the tumor cell, resulting in its death [84]. The lethal hit delivered by exosomes to targeted tumor cells is caused by the release of proteins and other molecules, including perforin, granzymes, and lysosomal enzymes. TCR activation stimulates the generation of CTL-derived exosomes, and similar work has demonstrated that the TCR/CD3 complex is present in the membrane of human CTL-derived exosomes [85].

CAR-T EVs and exosomes have shown lower toxicity potential compared to CAR-T cells. This makes them suitable as cell-free immunotherapy agents, which could simplify regulatory approval as they may not be classified as advanced therapy medicinal products. Additionally, CAR-T exosomes have low immunogenicity, allowing them to be used in a third-party setting as off-the-shelf products. Unlike parental cells, EVs and exosomes can easily penetrate tumor barriers, even in tumors with significant fibrotic reactions [86–88]. Studies have suggested that EV production and functionality can be improved by stimulating producer cells. Using CAR-T EV therapies in autologous CAR production eliminates the risk of reintroducing tumor cells into patients, which is a concern with CAR-T cell therapies [89]. It has been observed that residual tumor cells may inadvertently acquire CAR expression, leading to resistance to CAR-T cell therapy [90]. EVs derived from T-lymphocytes express TCR and proapoptotic molecules, giving them cytotoxic functions and antigen specificity. This suggests that CAR-T cell-derived EVs could effectively deliver proapoptotic signals to cancer cells. However, the presence of the CAR molecule on CAR-T cell-derived EVs is crucial for inducing specific cancer cell death, similar to their parental CAR-T cells [87]. The production of CAR-expressing exosomes can be enhanced by optimizing both the release of exosomes and the expression of CAR on their surfaces. TCR activation has been shown to increase the production of exosomes derived from CTLs. On the other hand, higher levels of CAR expression on exosomes can be achieved through various antigen stimulation techniques. These include using cell beads coated with the recombinant CAR target antigen or employing cells that express the CAR antigen. These strategies can help improve the efficiency and effectiveness of CAR-expressing exosomes production [89, 91].

Although the full understanding of CAR-T cell-derived exosomes is still under investigation, it is highly likely that they can deliver antigen-targeted cytotoxicity to cancer cells. This is because they contain targeting molecules on their surface that can bind to cancer cells, as well as cytotoxic molecules that can kill cancer cells.

### Direct attackers

CAR-T cell therapy has been less effective in treating solid tumors than lymphoid malignancies [92]. The tumor microenvironment in a variety of cancers can interfere with the anticancer function of CAR-T cells, in addition to the specificity of the tumor antigen used for CAR construction. The cell-to-cell interaction between CAR-T cells and tumor cells is a crucial need for this anticancer approach. However, solid tumors have a stroma-rich matrix that CAR-T cells must penetrate, unlike lymphoid malignancies. There are at least two ways in which the solid tumor microenvironment limits the effectiveness of CAR-T cells. First, CAR-T cell function may be restricted by aggressive tumor-mediated immunosuppression [93]. The second mechanism by which the tumor microenvironment can interfere with the efficacy of CAR-T cells is through functional modifications in T lymphocytes after their ex-vivo manipulation. This can make it difficult for them to penetrate the ECM of a solid tumor. Caruana et al. provided direct evidence to support the mechanism mentioned [94].

The primary constituents of the ECM, heparan sulfate proteoglycans, are degraded by the enzyme heparanase (HPSE), which was designed to express on CAR-T cells. The enhanced ability of HPSE CAR-T cells to diminish the ECM was shown to facilitate tumor T cell infiltration and eventually anticancer efficacy. However, due to their nanoscale size, exosomes produced by CAR-T cells can be used as direct cancer-attracter agents, which may facilitate CAR-mediated anticancer therapy. Exosomes produced from CAR-T cells must be able to act as direct attackers and be able to target particular antigen-targeted tumor locations in order to substitute CAR-T cells in anticancer therapies. In contrast to cell-based therapy, which requires therapeutic cells to actively move to the target site, cell-free exosomes can be delivered through biological fluids like blood circulation. Exosomes are able to cross biological barriers, including the blood-tumor barrier (BTB) and the blood-brain barrier (BBB). This is supported by the fact that tumor cell-derived exosomes have been found in bodily fluids. Additionally, intravenously administered exosomes are driven to the tumor by the tumor’s heightened retention effect and leaky vascular system [95]. Exosomes are reportedly passively transported throughout the body, but their target-oriented distribution is mostly influenced by the presence of tissue-specific receptors on their surface, which are derived from parent cells. Exosomes can kill cancer cells by transferring their contents to the target cell. This can happen through three distinct mechanisms. Firstly, via direct contact, exosomes can bind to specific receptors present on the surface of target cells, triggering signaling cascades that promote cell death. This interaction can activate apoptosis-inducing pathways or stimulate immune responses against the tumor cells. Secondly, exosomes can fuse with the plasma membrane of target cells, leading to the transfer of bioactive molecules, such as proteins and nucleic acids, into the cytoplasm. These transferred components can disrupt cellular homeostasis, initiate apoptotic pathways, or interfere with vital cellular processes. Lastly, exosomes can be internalized by target cells through endocytosis, allowing the release of their cargo into the intracellular space. The contents of the exosomes, including proteins, lipids, and nucleic acids, can then exert cytotoxic effects, trigger programmed cell death, or modulate cellular functions. These three mechanisms collectively demonstrate how exosomes can effectively mediate the killing of tumor cells and contribute to anticancer responses [96].

### Targeting specificity

An antibody-derived single-chain variable fragment (scFv) in the CAR structure controls the targeting specificity of CAR-T cells. The targeted attacking property is dependent on CD19-specific scFv in the treatment approach using CD19-CAR-T cells to treat CD19-positive hematological malignancies. The same antigen is expressed in normal organs or tissues, which is what causes the so-called “on-target, off-tumor” impact. Cellular membrane proteins, such as those involved in cell targeting, may be transported to exosomes during the biogenesis process. In a 24-hour period, 12% of the surface-bound peptide-MHC II complex in B cells is endocytosed, transported to MVBs, and released on exosomes, according to the research by Muntasell et al. [97].

It is likely that parent cells’ exosomes can pass down the targeting property. Numerous pre-clinical investigations have supported this viewpoint, while more research on exosomes made by CAR-T cells is still needed. Alvarez-Erviti et al.‘s direct proof of targeted exosomes was presented in their study [98]. They managed to produce mouse brain-targeted exosomes by genetically modifying dendritic cells to express Lamp2b, an exosomal membrane protein linked to the neuron-specific rabies viral glycoprotein (RVG) peptide. They then used these exosomes as delivery systems for siRNA to the brain. The availability of RVG, which acts as a ligand and precisely binds to the acetylcholine receptors in neurons, determines how exactly they are distributed to neurons following systemic delivery. Due to MSCs’ preference for primary and metastatic tumor sites, a mesenchymal stem cell (MSC)-mediated anticancer method was the subject of extensive research [99]. CAR-T exosomes offer several promising advantages over their cellular counterparts, including high target specificity, insensitivity to PD-L1 immunosuppression, and a reduced risk of inducing cytokine release syndrome. Similar encouraging results have been observed with exosomes derived from mesothelin (MSLN)-targeted CAR-T cells. These exosomes effectively targeted MSLN-positive and triple-negative breast cancer cells by secreting perforin and granzyme B, demonstrating their efficiency. Importantly, in vivo studies using BT-549 and MDA231-MSLN xenograft breast tumor models showed significant antitumor effects with low toxicity. These findings highlight the potential of CAR-T exosomes as a promising therapeutic approach for cancer treatment [100].

Tumor-derived biological factors and the existence of matching receptors in MSCs are necessary conditions for MSCs to be capable of tumor-directed homing. A continual source of cytokines, chemokines, and other inflammatory mediators, tumors are often described as “wounds that never heal.” However, MSCs express receptors for a variety of growth factors, such as PDGF and IGF-1, as well as chemokine receptors, including CCR2, CCR3, CCR4, and CCL5 [101]. It is important to note that all surface markers, signaling molecules, and cell adhesion molecules are also present in MSC-derived exosomes, which suggests that exosomes may adopt the homing pattern of the parent cell and acquire the same range of surface receptors and associated binding proteins as their parent cells.

This natural tropism indicates how essential it is to select the ideal cell source for exosome derivation for organ-specific therapeutic approaches. However, engineered CARs can offer non-HLA-restricted recognition of cell surface components and are not dependent on HLA for antigen processing and presentation [102]. CAR-T is therefore more broadly applicable to patient populations with diverse HLAs.

### Adapt to the complexities and changes of cancer

In addition to giving CAR-T cells antigen-specific targeting abilities, engineered CARs also enhance CAR-T cell growth and cytokine production. Cytokines are the most direct attackers and dramatically enlarged within CAR-T cells (primarily interleukines, either IL-2, IL-7, IL-15, or IL-21 [103]). However, because cancer tissue is profoundly heterogeneous and cancer cells are highly adaptable, which is the basis for drug resistance, no specific attacker or drug can effectively treat any specific cancer. Intrinsic resistance to chemotherapy exists in close to 50% of all cancer cases before drug treatment even begins, and acquired resistance occurs in a significant portion of the remaining 50% of cancer cases [104]. Due to the enormous heterogeneity and complex biology of cancer cells, which exhibit a wide range of individual variations, every attempt to overcome chemotherapy resistance to date has failed. This could be the cause of the limited success of CD19-CAR-T cell therapy in patients with CD19 + B-cell leukemia [105]. An ideal anticancer strategy should be diversified and capable of acting concurrently, taking into account the heterogeneity and variability of cancer. The anticancer medications can also be changed according to the unique circumstances of the patient. In fact, CAR-T cells and exosomes derived from CAR-T cells offer favorable platforms to carry out the aforementioned modifications.

Through CAR-T cells, additional anticancer medications can be added indirectly. Exosomes serve as a means of intercellular communication by transporting a variety of messages, including proteins, mRNAs, ncRNAs, and miRNAs, from the parent cell to the acceptor cell. Exosomes and parent cells both contain the vast majority of proteins, mRNAs, and miRNAs. Extracellular proteases and RNases prevent the degradation of the proteins and RNAs carried by exosomes, extending their half-lives and boosting their biological activity [106]. Exosomes isolated from DCs pulsed with tumor peptides were able to prime particular cytotoxic T lymphocytes in vivo and inhibit the growth of established murine tumors in a T-cell-dependent manner, according to preclinical studies in mice [107], demonstrating the manipulation of stimulation-induced signal transfer from parent cells to exosomes. It has shown before that HepG2 (a liver cancer cell) and ASPC-1 (a pancreatic cancer cell) were significantly cytotoxic when exposed to conditioned media (CM) from MSCs engineered with the anticancer gene TRAIL (MSC-CM) [108].

Exosomes produced by MSCs are likely crucial in the transport of anticancer agents from MSCs to cancer cells. Because TRAIL-bearing expression vectors were used for this anticancer gene engineering, TRAIL delivery via exosomes could take the form of cDNA, mRNA, or protein. Researches demonstrated using a myocardial infarction model that genetically modified MSCs cultured in vitro have CM with efficacy comparable to cell transplantation in preventing ventricular remodeling [109]. One or more cancer cell assailants may be indirectly loaded into exosomes produced by CAR-T cells during their biogenesis in ex vivo expanded CAR-T cells. Following that, multiple and additional attackers may be substituted depending on the patient’s therapeutic situation when combined with intrinsic T lymphocyte cytokines.

Additionally, anticancer medications can be directly loaded into exosomes made from CAR-T cells. Exosomes may contain both hydrophilic and lipophilic substances because they resemble liposomes, which also have an aqueous core and a bi-lipid membrane [110]. Exosome loading for hydrophilic molecules like mRNA, siRNA, and miRNA can be accomplished by momentary physical (e.g., electroporation) or chemical (e.g., lipofection) disruption of the exosome membrane. Exogenous siRNA was directly loaded into DC-derived exosomes by electroporation in Alvarez-Erviti et al.‘s study, which caused strong mRNA and protein knockdown of the targeted genes in target cells [111]. However, a brief period of direct co-incubation can load exosomes with hydrophobic molecules.

Intracellular anticancer gene products are easier to use when delivered via exosomes, which act as nano-carriers. P53, Myc, and phosphatase and tensin homolog (PTEN) are examples of intracellular mechanisms used by the majority of anticancer gene products to affect tumor cells. In the field of MSC-mediated gene therapy, MSCs are used as a vehicle to deliver anticancer genes. The intracellular anticancer gene products need to be produced in MSCs and secreted into extracellular space, and then they need to penetrate nearby tumor cells passing through the biological membrane, which is a natural barrier for most macromolecules, including peptides and proteins. Therefore, the construct of the associated expression vectors typically contains a leading sequence and a transacting activator of transcription (TAT) [112]. Exosomes, however, have been demonstrated to pass the plasma membrane in order to deliver their cargo to the recipient cells. For instance, exosomes derived from DCs can deliver MHC I and II complexes loaded with peptides to other DCs and CD4 + T cells to control the immune response [10, 104]. mRNA and protein from the parent cell’s cytoplasm can be transferred directly into the cytoplasm of the recipient cell, avoiding the biological membrane. Theoretically, through exosome biogenesis, anticancer gene-engineered MSCs could deliver anticancer gene products in all conceivable forms, including cDNA, mRNA, and protein. This may account for the lack of distinction between TRAIL-secreting and TRAIL-nonsecreting engineered MSC-induced cancer cell death. Evidently, exosomes will expand and improve cell-based gene therapy for the treatment of cancer with the proper application. Some of Therapeutic potential of CAR T cell-derived exosomes on tumor cells are summarized in Table 2.

**Table 2:** Therapeutic potential of CAR T cell-derived exosomes on tumor cells

Target molecules	Type of cancer	Anti-tumor structure	Mechanism	Reference
HEGFR	Breast cancer	human EGFR-specific CAR	Direct attacker	[5, 119]
HER2	Breast cancer	HER2-specific CAR	Direct attacker	[5, 119]
MSLN	Triple-Negative BC (TNBC)	Granzyme B, Perforin	Inhibit tumor growth [71, 118, 120]	[71, 118, 120]
	Mesothelin-positive Lung cancer	Granzyme B, Perforin(PTX-based chemotherapy, Lip-CExo@PTX)	Inhibit tumor growth/Enhance the antitumor effects	[121, 122]
PD-L1	blocked PD-L1 on the tumors to	Granzyme B, Perforin(PTX-based chemotherapy, Lip-CExo@PTX)	T cell exhaustion avoidance/boost the PTX-induced immunogenic cell death	[121, 122]

Abbreviation: CAR: Chimeric antigen receptor, HEGFR: Human epidermal growth factor receptor, HER2: Human epidermal growth factor receptor 2, MSLN: Mesothelin, PD-L1: Programmed death-ligand 1, PTX: paclitaxel

## Conclusion

Utilizing T lymphocytes that have been genetically altered to express CARs, CAR-based adoptive immunotherapy can elicit immune responses that are both T-cell-mediated and specific for the antigen being targeted. After being infused into the body, CAR-T cells function as “living drugs” that continuously launch cytotoxic attacks against the intended malignant cells. Their own lifespan and future in vivo expansion determine how long their potent tumor-killing ability will last. However, it is not possible to adequately control the amount of cytokine release from CAR-T cells or the state of these cells’ in vivo expansion, which is a potential source of adverse events like CRS, cytokine storms, and “on-target, off-tumor” responses.

Exosomes made from CAR-T cells have great therapeutic potential because they can take the place of CAR-T cells, which target tumor cells. Exosomes can serve as a direct adversary for cancer therapy in place of CAR-T cells, with obvious advantages, due to their cell-free nature and biological characteristics. First, using exosomes as “off-the-shelf” reagents makes CRS manageable. The appropriate use of exosomes also paves the way for the application of CAR-T technology for the treatment of solid tumors, including the targeting of tumors in particular regions, like glioblastoma, due to their nanoscaled size [113]. Third, in addition to the intrinsic cytokines from CAR-T cells, extra attackers can be added to CAR-T cell-derived exosomes to prevent potential resistance, and fourth, the combined and/or alternate use of these two platforms (i.e. CAR-T cells and CAR-T cell-derived exosomes) will undoubtedly strengthen the application for CAR-based cancer therapy.

**Fig. 3:**
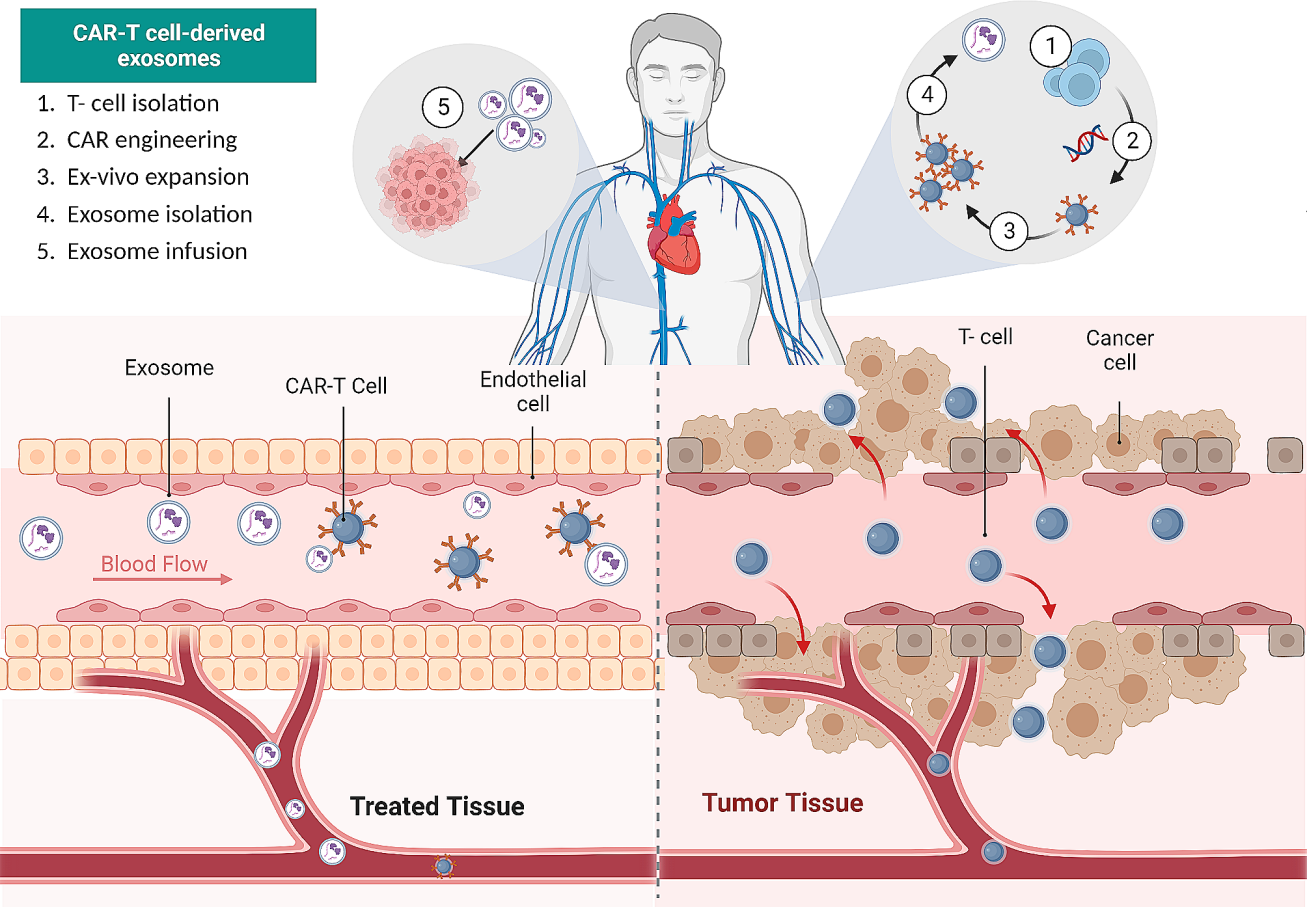
Progress in target identification, improving tumor penetration, toxicity management, and investigating combination therapies provide optimistic prospects for the successful application of CAR-T cell therapy against solid tumors. These advancements bring us closer to personalized and precise cancer treatments. A cancer patient’s peripheral blood sample is collected to isolate total nucleated cells. These cells are then cultured in the presence of T cell stimulators, such as CD3 and CD28 antibodies. The selected CD8 + T cells are genetically modified by introducing chimeric antigen receptors (CARs) through either viral or non-viral transfection techniques. The CAR-engineered T cells are further expanded in. Exosomes derived from the CAR-T cells are isolated from the culture. Finally, the isolated exosomes are infused back into the same patient after preconditioning with chemotherapy

According to the proposed method for using CAR-T cell-derived exosomes in clinical settings, shown in Fig. 3, T cells from the peripheral blood of cancer patients are collected, CARs are either virally or non-virally inserted into the T cells, the CAR-engineered T cells are expanded ex vivo, exosomes are isolated, and the patient is then given the exosomes. Based on the unique properties of the exosomes, such as size (30–150 nm), density (1.1–1.18 g/ml), and evolutionary conserved set of protein molecules (CD9, CD63, CD81, Alix, and Tsg101), CAR-T cell derived exosomes can be isolated from culture medium using a variety of techniques, including ultrafiltration, ultracentrifugation, and affinity capture on antibody-coupled magnetic beads [114].

Before using CAR-T cell-derived exosomes in any clinical setting, it is definitely warranted to conduct preclinical studies to characterize them.

In conclusion, a promising therapy for the treatment of cancer is CAR-based T-cell adoptive immunotherapy. As a cutting-edge and effective therapeutic approach, it is running into some flaws or restrictions due to the complexity and volatility of cancer. Exosomes derived from CAR-T cells may replace CAR-T cells to act as ultimate attackers as an extension of this effective modality, thereby overcoming some of the shortcomings in current treatment models. This effective treatment will become more functional with the appropriate integration of cellular and exosomal platforms, and should move us one step closer to the creation of a novel targeted cancer treatment option.

Also, as future development, we can refer to selectively loading required mRNA or control target gene loading within exosomes. Recent studies propose strategies for selectively loading mRNA or controlling target gene loading into exosomes. This includes engineering donor cells, modifying exosome surfaces, and using synthetic nanoparticles [115–117]. Future advancements aim to enhance the targeting and incorporation of specific transcripts, improve stability, and optimize loading efficiency. Innovative gene delivery systems and identifying specific markers on exosomes and recipient cells can enable precise cargo delivery. These developments hold promise for therapeutic applications like precision medicine and gene therapies.

## Data Availability

All data is available in this article.

## Abbreviations

CAR-T: Chimeric antigen receptor-T
CRS: Cytokine release syndrome
ESEs: Early sorting endosomes
LSEs: Late sorting endosomes
MVBs: Multivesicular bodies
TME: Tumor microenvironment
TAAs: Tumor-associated antigens
GBM: Glioblastoma
OVs: Oncolytic viruses
ICIs: Immune checkpoint inhibitors
GVHD: Graft-versus-host disease
EVs: Extracellular vesicles
CTL: Cytotoxic T lymphocytes
HPSE: Heparanase
BTB: Blood-tumor barrier
BBB: Blood-brain barrier
scFv: Single-chain variable fragment
RVG: Rabies viral glycoprotein
MSC: Mesenchymal stem cell
CM: Conditioned media
TAT: Transacting activator of transcription
